# Case report: Successful surgical resection of an intracranial frontal lobe dermoid cyst in a cat

**DOI:** 10.3389/fvets.2025.1512097

**Published:** 2025-02-14

**Authors:** Yukiko Nakano, Yuta Nozue, Hiromi Hazeyama, Tokio Matsunami, James Chambers, Kazuyuki Uchida, Yui Kobatake

**Affiliations:** ^1^Department of Neurosurgery, ER Hachioji Advanced Animal Medical and Critical Care Center, Hachioji, Japan; ^2^Animal Medical Center, Gifu University, Gifu, Japan; ^3^Matsunami Animal Hospital, Nagoya, Japan; ^4^Laboratory of Veterinary Pathology, Graduate School of Agricultural and Life Sciences, The University of Tokyo, Tokyo, Japan

**Keywords:** intracranial dermoid cyst, frontal lobe, epileptic seizure, surgical management, cat

## Abstract

**Introduction:**

Intracranial dermoid cysts (IDCs) are rarely observed in veterinary medicine, and reports regarding treatment strategies for feline IDCs are severely lacking. This report describes the surgical management of epileptic seizures caused by IDCs in a cat.

**Case presentation:**

An 8-year-old, spayed, female American Shorthair cat presented with epileptic seizures. The epileptic seizures, which had developed at the age of 5 years, had been controlled by phenobarbital administration. At 8 years old, the cat contracted acute hepatitis, prompting a switch from phenobarbital to other antiseizure medications. This drug switch caused an increase in the frequency of epileptic seizures. Magnetic resonance imaging (MRI) revealed a dermoid cyst as a heterogeneous intensity mass on T2-weighted images, without falx cerebri displacement. The preoperative seizures occurred more than three times a day (cluster seizures), even though the cat was administered multiple antiseizure medications. The seizures ceased after surgical removal of the dermoid cyst. The cat did not experience seizures for 14 months after surgery, even with discontinuation of antiseizure medications.

**Conclusion:**

In cats, surgical removal of frontal lobe IDCs may effectively control epileptic seizures without fatal complications, thus potentially leading to a great prognosis.

## 1 Introduction

Intracranial dermoid cysts (IDCs) are rare in veterinary medicine, although they have been occasionally reported (1–3). IDCs are congenital lesions characterized as benign mass lesions in the cranial cavity containing keratinaceous debris and adnexa, which include hair follicles, sebaceous, apocrine, and sweat glands covered by squamous epithelium (3). Dermoid cysts originate from ectoderm and mesoderm remaining in the embryonic fusion plane (4). Intracranial extension of nasal dermoid cyst in a cat has been also reported (5). Reports of surgical management of a dog treated with ventriculoperitoneal shunt (3) and a cat treated with surgical removal (5) are available; however, to date, there are no reports of successful surgical treatment of IDCs in cats worldwide, and information on treatment strategies for feline IDCs is severely lacking. In this report, we describe the clinical symptoms, diagnostic findings, surgical procedures, and outcomes of a cat with frontal lobe IDCs.

## 2 Case presentation

### 2.1 Signalment and history

A 5-year-old spayed female American Shorthair cat presented to a local veterinary hospital with a single epileptic seizure (day 1). Computed tomography revealed an intracranial 7.4 mm × 8.6 mm × 13 mm mass located in the frontal lobe region. The primary veterinarian suggested surgical intervention for the mass; however, the owners declined and elected to manage the seizures. Phenobarbital (3 mg/kg, PO, q12h, Phenobal tablets 30 mg, Daiichi Sankyo, Inc., Tokyo, Japan) was administered, and the patient was seizure-free for 3 years. However, 3 weeks before visiting the Animal Medical Center of Gifu University (day 872), when the cat was 8 years old, she developed acute hepatitis. The local veterinarian discontinued phenobarbital administration and switched to zonisamide (3 mg/kg, q12h, PO, CONSAVE tablets 25 mg, DS Pharma Animal Health, Osaka, Japan) due to the deterioration of hepatic function. After this switch, cluster seizures were observed, and levetiracetam (20 mg/kg, q8h, PO, E Keppra tablets 250 mg, UCB Japan, Tokyo, Japan) and gabapentin (5 mg/kg, q8h, PO, GABAPEN tablets 200 mg, Pfizer Japan, Tokyo, Japan) were administered. The additional antiseizure medications resulted in 3 seizure-free days. The acute hepatitis was treated with enrofloxacin (5 mg/kg, PO, q24h, Enroclear 50 mg tablet; Kyoritsu Seiyaku Corporation, Tokyo, Japan), amoxicillin (11 mg/kg, PO, q12h, Amoxiclear 100 mg tablet, Kyoritsu Seiyaku Corp.), and prednisolone (1 mg/kg, q24h, PREDONINE 5 mg; Shionogi Pharma Corp., Osaka, Japan). This treatment improved her condition and elevated hepatic enzyme levels.

**Figure 1:**
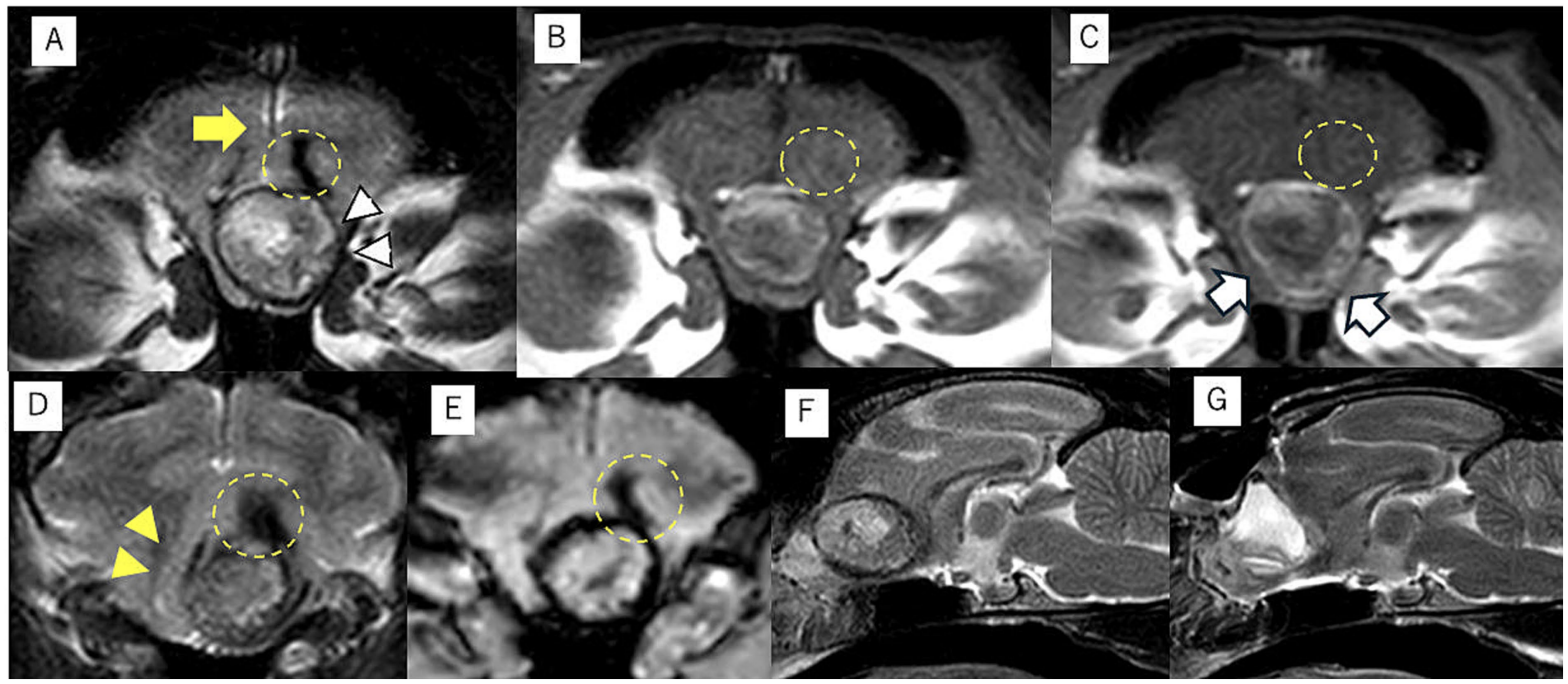
Magnetic resonance imaging of intracranial dermoid cysts (IDCs). The transverse T2-weighted image demonstrates a mass lesion with heterogeneous intensity at the midline. The center of the mass lesion is hyperintense, and the periphery is isointense to hypointense (white arrowhead) **(A)**. This transverse T1-weighted image shows the center is hypointense, and the periphery is isointense to hyperintense **(B)**. The edge of IDCs is mildly contrast enhanced in the transverse postcontrast T1-weighted image (white arrow) **(C)**. The falx cerebri midline shift is not observed at the middle level of the IDCs (**A**, yellow arrow). However, the brain parenchyma has shifted to the left side at the caudal slice of the IDCs in the transverse T2-weighted image (**D**, yellow arrowhead). The transverse T2-weighted images showed a hypointense area in the white matter adjacent to the IDCs without edema, and it was demonstrated as an isointense area without contrast enhanced in T1WI (yellow dotted circle) **(A–D)**. This lesion was also detected as a hypointense area on the T2* transverse image (**E**, yellow dotted circle). The preoperative **(F)** and postoperative **(G)** midsagittal planes of T2-weighted images demonstrated that IDCs have been completely removed after surgery, and the saline used during the surgery is accumulated in the extraction cavity **(G)**.

The patient presented to the Animal Medical Center of Gifu University on day 889 for further examination and treatment of the intracranial mass and epileptic seizures. At presentation, the vitals were normal, and the physical examination was unremarkable. All items of the complete blood count were within the normal range, and serum biochemistry was mostly normal except for mildly increased alanine aminotransferase (ALT; 107 IU/L, reference interval: 22–84 IU/L). Thoracic and abdominal radiography was also normal. Neurological examination revealed the menace response reduced bilaterally. The clinical history and neurological examination suggested that the neurolocalization was forebrain. Magnetic resonance imaging (MRI; 3.0-T magnet, Achieva 3.0 T, Philips Japan, Tokyo, Japan) revealed a mass lesion (8.9 mm × 10 mm × 13 mm) in the left frontal lobe. Transverse, sagittal, and dorsal T1-weighted images (T1WI; TR, 2,000 ms; TE, 8) and T2-weighted images (T2WI; TR, 3,600; TE, 90) and T2-w fluid-attenuated inversion recovery (FLAIR; TR, 10,000 ms; TE, 100 ms) were obtained. Transverse T2*-weighted images (T2*WI; TR, 780 ms; TE, 18 ms) were also acquired. Postcontrast T1WI was obtained following intravenous administration of gadodiamide contrast medium (0.1 mmol/kg, Omniscan; GE Healthcare, Tokyo, Japan). Postcontrast T1WI was acquired in the transverse, sagittal, and dorsal planes. All slice thicknesses were 2 mm. The lesion showed heterogeneous signal intensity on T2WI, which was mainly isointense, and showed internal hyperintensity and hypointensity in the remaining part compared with the normal brain gray matter (Figures 1A,D,E). The margins were demarcated. The T1WI demonstrated hyperintensity at the margins and heterogeneous hyperintensity inside the mass (Figure 1B). The postcontrast T1WI revealed slight contrast enhancement at the margin of the mass (Figure 1C). The white matter adjacent to the mass appeared as the hypointense region in T2WI and T2*WI and isointense without contrast enhancement in T1WI. However, peripheral brain edema was not observed (Figures 1A,D,E). Although the lesion mildly compressed the brain parenchyma, displacement of the falx cerebri was not observed (Figures 1A,D). The abnormality of the cribriform and nasal cavity was not detected as well. The cerebrospinal fluid (CSF) could not be obtained. Considering the case signalment, the clinical history, and the MRI findings, the differential diagnosis included anomalous (such as dermoid cyst, epidermoid cyst, and subarachnoid cyst), infectious or inflammatory (such as cholesterol granuloma, toxoplasma gondii granuloma, brain abscess, and cryptococcal granuloma), and neoplastic diseases (meningioma and lymphoma). However, none of those diseases were completely consistent with the findings of this case.

Presumably, the epileptic seizures would be difficult to control with medical therapy despite the administration of multiple antiseizure medications hereafter because cluster seizures were observed again the day after the first presentation at the Animal Medical Center of Gifu University (day 875). Moreover, the cluster seizures were observed almost every day after day 875. The dose up of the antiseizure medications seemed to be required to control the seizures with medicine. However, the effect of increasing the dose of medicines would not be immediate. The owners decided to have the cat undergo surgical removal of the mass, which was performed on day 907.

### 2.2 Surgical procedure

**Figure 2:**
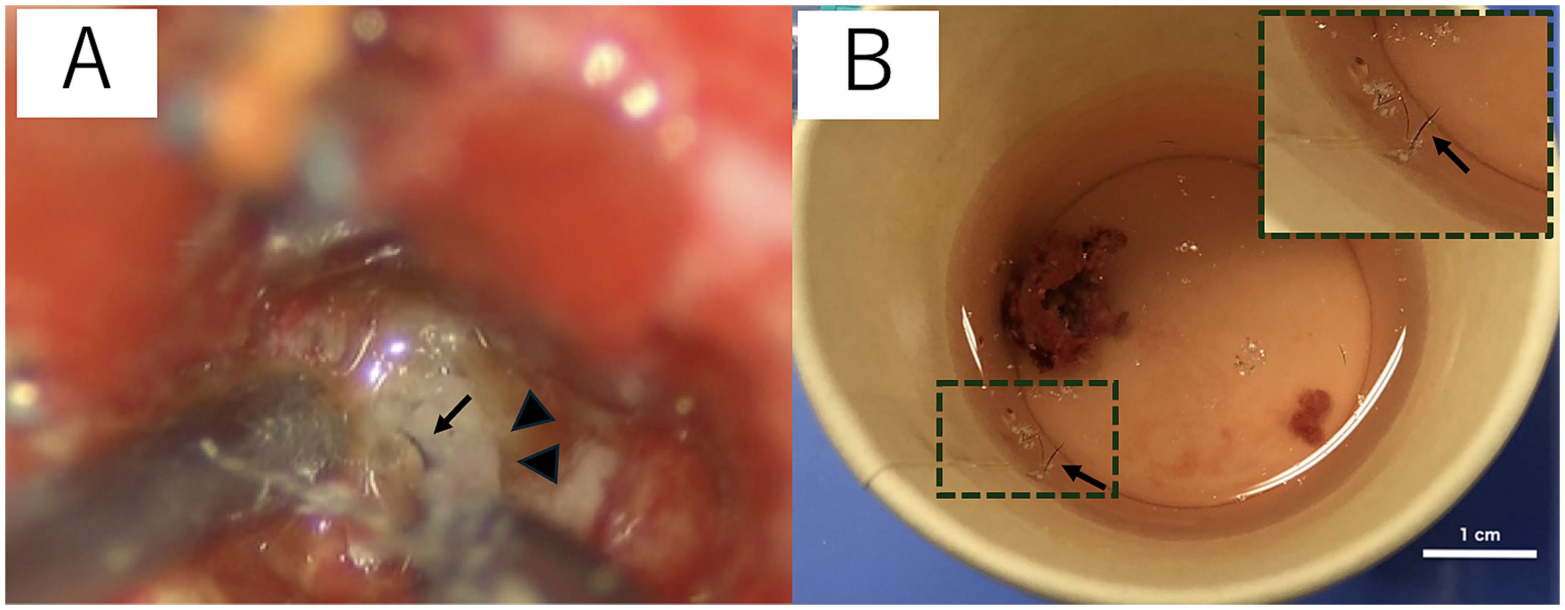
Surgical microscopic image **(A)** and the macroscopic image of the resected intracranial dermoid cysts (IDCs) soaked in saline **(B)**. IDCs contain hair (black arrow) and keratin (arrowhead) **(A)**. The hair is separated from the IDC contents and is floated in saline **(B)**. The image in the top right rectangular dashed frame is a magnified image of hair suspended in saline **(B)**.

The cat was induced with alfaxalone [2.5 mg/kg, intravenous (IV) to effect, Alfaxan multidose; Meiji Animal Health, Kumamoto, Japan] and maintained with sevoflurane with 40% 0_2_ and alfaxalone, constant rate infusion (CRI) (3–4 mg/kg/h). She was also administered cefazolin sodium (20 mg/kg, IV, Cefamezin Alpha; LTL Pharma, Tokyo, Japan), maropitant citrate (1 mg/kg, SC, Cerenia, Zoetis Japan, Tokyo, Japan), famotidine (0.5 mg/kg, IV, Gaster injection 20 mg, LTL Parma), and prednisolone (1 mg/kg, SC, prednisolone injection; Kyoritsu Seiyaku, Tokyo, Japan) as pre-anesthetic medication. The patient was positioned in ventral recumbency on a vacuum bean bag. Adequate intraoperative analgesia was achieved with fentanyl (20–30 mcg/kg/h CRI, fentanyl injection 0.5 mg, Terumo, Tokyo, Japan). Dopamine hydrochloride (1–13 mcg/kg/h, CRI, dopamine hydrochloride 100 mg IV infusion, Nipro, Osaka, Japan) was used to control intraoperative blood pressure. Craniotomy was performed using a transfrontal sinus approach. After craniotomy, the dura mater was incised, and the mass was approached through a cortical incision in the left frontal lobe. When the hard mass covered by a capsule was visually recognized, the capsule was incised, and internal decompression of the mass was performed. A large amount of keratinized material, including hair, was collected from inside the mass (Figure 2). Some of the contents were subjected to bacterial culture testing. The mass was internally decompressed, and the capsule was separated from the surrounding brain parenchyma. The brain parenchyma around the mass seemed normal. The brain parenchyma and capsule were easily separable. As the capsule of the mass was completely continuous with the falx cerebri, the capsule of the mass was excised along with the falx cerebri. The rostral part of the mass was attached to the lamina cribrosa; therefore, it was cauterized and sectioned using bipolar on the rostral side as far as possible. After the irrigation of the extraction cavity with sterile saline, the dural replacement was performed using the fascia. The left temporal fascia was collected and divided into two pieces: one was attached to the cranial cavity side of the cribriform plate, whereas the other was used for dural reconstruction. Fibrin glue (Beriplast P Combi-Set Tissue adhesion, CSL Behring, Tokyo, Japan) was used to adhere the dura mater to the fascia graft. The skull was reconstructed using excised bone flaps, titanium screws (U-CMF AXS Self-drilling Screw 3 mm; Stryker, Tokyo, Japan), and titanium plates (Dynamic Mesh 0.6 mm, Stryker). The bone flap was returned to the originated location and stabilized with tiny, thin titanium plates and screws on four sides instead of sutures. MRI examination was performed immediately after surgery, and complete removal of the mass was confirmed (Figures 1F,G).

**Figure 3:**
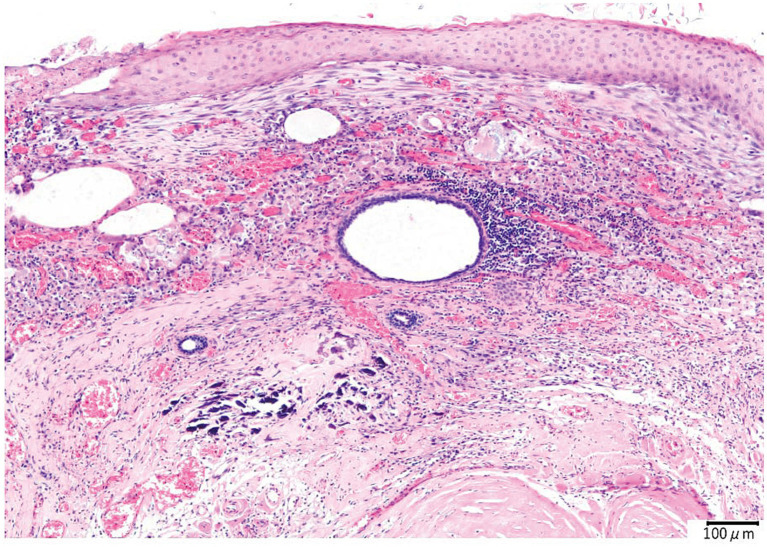
Histopathological image of the intracranial dermoid cyst. The cyst is lined by the stratified squamous epithelium. Concentric layers of exfoliated keratin are observed in the cyst. Lymphocyte and macrophage infiltrates around the cholesterol clefts and hair are also observed. Hemorrhage, hemosiderin-laden macrophages, and multinucleated giant cells are also observed in the inflammatory focus. At the edge of the inflammatory foci, the proliferation of collagen fibers, angiogenesis, and calcification is revealed.

The histopathological findings were the cyst wall was formed by the stratified squamous epithelium, and the cyst contained exfoliated keratin that was arranged concentrically, hair, and inflammatory cells such as lymphocytes and macrophages but did not include the skin adnexa (Figure 3). An epidermoid cyst was suspected by the postoperative histopathological examination; however, the finding that the mass contained hair was not consistent with the feature of epidermoid cysts. The bacterial culture test results were negative. The mass was made a diagnosis of IDCs based on the comprehensive evaluation of the clinical and histopathological findings.

### 2.3 Postoperative clinical course

No postoperative neurological deficits were observed, and the patient was discharged 1 day after surgery and administered an antibacterial drug (cephalexin, 17 mg/kg, PO, q12h, Cefaclor tablet 75; Kyoritsu Seiyaku Corp.) and antiseizure medications, including zonisamide (6 mg/kg, PO, q12h) and levetiracetam (15 mg/kg, PO, q12h).

Two weeks after surgery, the surgical wound became infected because the cat rubbed the surgical wound against the wall. Potassium clavulanate amoxicillin hydrate (15 mg/kg, PO, q12h; Augmentin Combination Tablets; GSK, Tokyo, Japan) was administered, which resulted in complete scar closure.

**Figure 4:**
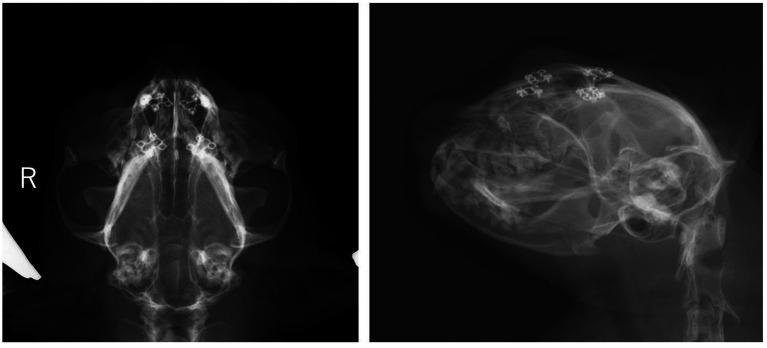
Radiographs of head on the day 1,377. Four titanium plates and eight screws are used to stabilize the outer plate of the frontal sinus. Alternation in opacity around the implants or implant migration is not observed.

The epileptic seizures disappeared after surgery, and antiseizure medications were tapered off and completely discontinued on day 964. On day 1,337 (approximately 14 months after surgery), the cat presented to the local clinic for a health checkup. The patient was making good improvement without seizures and other neurological deficits, even after the discontinuation of the antiseizure medications. Radiographs showed no abnormalities in the titanium plates and screws as well (Figure 4).

## 3 Discussion

To our knowledge, this was the first report of a dermoid cyst in the frontal lobe of a cat that was successfully treated. Three case reports of IDCs have been presented in veterinary medicine (2, 3, 5). As clinical symptoms, a dog with IDCs in the fourth ventricle had head tilt, hind limb ataxia, aggressive behavioral change, and a 7-month history of progressive abnormal behavior such as avoiding exercise and bumping into obstacles (3). Another dog with IDCs in the left cerebellar peduncle presented head tilt and a 3-year history of episodes of hind limb weakness and ataxia (2). One case report described a cat with a nasal dermoid cyst that extended to the calvarium and contacted the left olfactory bulb and frontal lobe (5). In this case, the clinical signs were symptoms of the forebrain, including focal epilepsy. Reported clinical signs of infratentorial IDCs include hind limb ataxia and vestibular dysfunction (2, 3). Fourth ventricle IDCs can also present with signs involving the forebrain, including increased aggression and visual impairment, although these clinical signs suggest secondary obstructive hydrocephalus (3). The forebrain signs for fourth ventricle IDCs in the previous report did not include epileptic seizures but included mentation abnormalities, whereas those of IDCs affected the frontal lobe in both of the cases previously reported and our case included only epileptic seizures. The differences in forebrain signs between the secondary obstructive hydrocephalus and the fourth ventricle and frontal lobe IDCs may include increased aggression and behavior changes.

The intracranial cyst in this case contained not only the squamous epithelium but also a small amount of hair. Dermoid cysts have been reported as intracranial mass lesions which commonly contain hair in human medicine, whereas epidermoid cysts, which have similar pathological features to dermoid cysts, contain only the squamous epithelium. The difference between dermoid cysts and epidermoid cysts is the presence of not only hair but also the skin adnexa, including dermal elements such as hair follicles, sebaceous glands, and sweat glands in humans (6). IDCs sporadically have been reported in veterinary medicine, including one feline case (3, 5). The MRI findings of the previous feline case with dermoid cyst were heterogeneous hyperintensity on both T1WI and T2WI, rim contrast enhancement, located midline, and nasal cavity lesion extending through the cribriform plate and into the calvarium (5). These features are remarkably similar to our case; however, a nasal lesion connected to the intracranial mass was not detected by imaging modalities. The MRI findings and the presence of hair suggested it was a dermoid cyst, although the histopathological examination could not have detected the skin adnexa. Our case was eventually diagnosed with IDCs based on an MRI and the presence of hair in the cyst. The definitive reason for the cyst containing hair was unclear. One of the possibilities is that the intracranial mass connects to the invisible small nasal dermoid cyst resulting in the intracranial mass being made as the cyst that contains hair. Monitoring with imaging modalities such as CT and MRI may be necessary hereafter whether any nasal cavity lesions are detected.

Common intracranial mass lesions are accompanied by a mass effect (7). Usually, mass lesions are also unevenly distributed on either the left or right side (7). However, in this case, no obvious displacement of the falx cerebri was observed, whereas displacement of the brain parenchyma due to the mass effect occurred. This is consistent with the pathophysiology of this condition, in which the epithelial tissue remains in the neural tube when the brain is formed during neural tube closure in the embryonic period (8). In human medicine, IDCs are already known to be predominantly observed near the midline structure (8). The midline location of the mass and the absence of displacement of the falx cerebri might be one of the characteristic findings in frontal lobe IDCs in veterinary medicine as well. A mass effect in the brain parenchyma may be caused by IDC growth after birth, although there was no evidence of significant IDC size increase between the CT images on day 1 and MR images on day 889 in this case. The size increase might have been associated with the onset of the epileptic seizure, if it had occurred before the onset of the epileptic seizure. The alternation in the IDC size could not have been perceived because clinical symptoms other than epileptic seizures were not observed.

There was no significant change in the size of the IDCs between days 1 and 889. It seemed to be slightly larger in vertical and horizontal length; however, the difference is a minor change that is potentially affected by the difference in imaging modality. This finding suggested that the cause of increased seizure frequency was not IDC enlargement. The other possible cause could be withdrawal seizures. Administration of the initial antiseizure medication, phenobarbital, was discontinued rather than tapered off. This sudden change in antiseizure medication could result in withdrawal seizures. To prevent withdrawal seizures, the dose of the antiseizure medications is recommended to be decreased by 20% or less on a monthly basis (9). However, phenobarbital discontinuation was inevitable because of severe hepatic dysfunction in this case. Readministration of phenobarbital in this case also was not recommended, even though cluster seizures were observed with other antiseizure medications, such as zonisamide, levetiracetam, and gabapentin. The seizures eventually completely ceased after surgery; this result suggests that the cause of the seizures is potentially associated with the presence of IDCs. The remaining possibilities of the cause could be the inflammation and hemorrhage inside or outside the IDCs. Histopathological examinations revealed chronic inflammation and hemorrhage in the IDCs. T2WI and T2*WI on MRI showed a hypointense region in the white matter adjacent to the IDCs, which could be a chronic hemorrhage outside of the IDCs (10). T2* WI hypointense lesions include hemorrhage, mineralization, gas, fibrous tissue, and iron deposits (10). Mineralization, gas, and fibrous tissue were hypointense in both T2WI and T1WI, which is not consistent with our case. Iron deposits including hemorrhage (deposits of hemosiderin) could be the lesion. However, intraoperative findings did not reveal hemorrhage in the white matter around the IDCs. It is possible that the lesion was small, chronic, and a mild change, and it was located inside the normal brain parenchyma, so the surgeon could not recognize the alternation. The IDCs may have elicited inflammation of brain tissue surrounding the IDCs; however, evidence could not be identified from preoperative MRI and intraoperative findings. CSF analysis could have been useful for detecting intracranial inflammation, although CSF was not collected in this case.

Surgical intervention for IDCs has been reported in a cat (5) and a dog (3). The neurological signs, including focal seizure activity, of the cat who had surgical resection of IDCs continuous with a nasal lesion at the frontal lobe temporarily improved, but rhinosinusitis with suspected local meningitis of the left olfactory bulb was observed as the complication more than 8 months after surgery, and the seizure activity relapsed (5). In our case, the mass lesion was found only in the cranial cavity, so the destruction of the cribriform plate was not observed even after surgery. It was suggested that the infection of the sinus and meninges was not observed because of the intact cribriform plate in our case. If the IDCs are connected to the nasal lesion, the complications of the infection of the sinus and meninges should be monitored, although the risk of such complications may be lower for the IDCs localized only to the frontal lobe. However, the skin adnexa, including hair follicle, was not observed in the pathological tissue of our case. This finding indicates the possibility that there is a residual lesion around the resection cavity. The prognosis of this case was excellent at 14 months after surgery, although longer follow-up and MRI scans are required because the possibility of recurrence and infection is still present. The dog with IDCs in the fourth ventricle and secondary hydrocephalus was treated with a ventriculoperitoneal shunt. The neurological deficit improved temporarily; however, the dog was euthanized because of the deterioration of neurological symptoms 4 weeks after surgery. To our best knowledge, there is no report of the resection of the medullary IDCs, although there are two reports of direct surgical intervention for the fourth ventricle of the intracranial epidermoid cyst, which has a similar pathology to IDCs. The medulla oblongata was injured by surgical invasion in the dog who underwent surgical resection of an intracranial epidermoid cyst in the fourth ventricle (11). In another previous report, a suboccipital approach for intracranial mass lesions and abnormal results on preoperative neurologic examination, including head tilt and abnormal mentation, were identified as risk factors associated with death (12). In addition, the odds ratio of death with a suboccipital approach increased in dogs with brain stem tumors in this report (12). Our feline case, which had frontal lobe IDCs, did not develop any fatal complications except for surgical wound infection as a minor complication. This result suggests that the risk factors associated with the surgical removal of IDCs are similar to other intracranial masses, including epidermoid cysts and brain tumors. The decision to perform surgical intervention ought to be made with caution if IDCs or epidermoid cysts are located in the infratentorial region, whereas the surgical resection of frontal lobe IDCs could be a relatively safe and reasonable treatment option.

Therefore, surgical resection of frontal lobe IDCs should be considered as a therapeutic option, especially if clinical symptoms are difficult to control with medicinal therapy. In our case, the macroscopic lesion of IDCs was completely removed, and the clinical signs have not recurred over 1 year after the surgery.

## Data Availability

The original contributions presented in the study are included in the article/supplementary material, further inquiries can be directed to the corresponding author.

## Supplementary material

Supplementary Video S1 The video shows intracranial surgical manipulation for extraction of dermoid cyst. The capsule of dermoid cyst was observed after incision of the dura matter and the cerebral cortex of the left frontal lobe. The capsule was made incision, white keratinized material and hair were recognized in the capsule. Internal decompression of the cyst was completed, and the capsule was easily detached and removed from the normal brain parenchyma.
